# Supplementary material for: Age-dependent effects of protein restriction on dopamine release
Source: Neuropsychopharmacology. 2020 Jul 31;46(2):394–403. doi: 10.1038/s41386-020-0783-z (PMC7852901; doi:10.1038/s41386-020-0783-z)
Supplement: Supplementary file 1 — Supplemental Tables and Figures [file 41386_2020_783_MOESM1_ESM.docx]

**Supplementary Table 1.** Macronutrient composition of the control chow and protein restricted diet.

|  | **Control chow diet**  **(Teklad global #2918)** | | **Protein restricted diet**  **(Research Diet D15100602)** | |
| --- | --- | --- | --- | --- |
|  | g (%) | kcal (%) | g (%) | kcal (%) |
| **Protein** | 18 | 24 | 5 | 4 |
| **Carbohydrate** | 62 | 58 | 76 | 74 |
| **Fat** | 7 | 18 | 10 | 22 |

**Supplementary Table 2**. Macronutrient breakdown of average daily food intake per cage (in g) or normalized to rats’ body weight (g/kg body weight) for each diet condition.

|  | | **Rats (Cages)** | **Total food (per cage)** | **Macronutrient** | | |  |
| --- | --- | --- | --- | --- | --- | --- | --- |
|  |  |  |  | **Protein** | **Carbohydrate** | **Fat** |  |
| **Adults** | **NR** | 8(4) | 27.7 ± 0.7 | 5.1 ± 0.1 | 17.3 ± 0.4 | 1.9 ± 0.1 | g |
|  |  |  | 69.7 ± 2.7 | 13.0 ± 0.5 | 43.5 ± 1.7 | 5.0 ± 0.2 | g/kg body weight |
|  | **PR** | 7(4) | 28.7 ± 0.4 | 1.4 ± 0.02 | 21.8 ± 0.3 | 2.9 ± 0.04 | g |
|  |  |  | 69.9 ± 2.7 | 3.5 ± 0.1 | 53.1 ± 2.0 | 7.0 ± 0.3 | g/kg body weight |
| **Adolescents** | **NR** | 6(3) | 22.7 ± 0.8 | 4.2 ± 0.1 | 14.2 ± 0.5 | 1.6 ± 0.05 | g |
|  |  |  | 130.1 ± 3.0 | 24.2 ± 0.6 | 81.2 ± 1.9 | 9.4 ± 0.2 | g/kg body weight |
|  | **PR** | 7(3) | 14.5 ± 1.7 | 0.7 ± 0.08 | 11.0 ± 1.3 | 1.4 ± 0.2 | g |
|  |  |  | 117.8 ± 9.4 | 5.9 ± 0.3 | 89.5 ± 4.1 | 11.8 ± 0.5 | g/kg body weight |

**
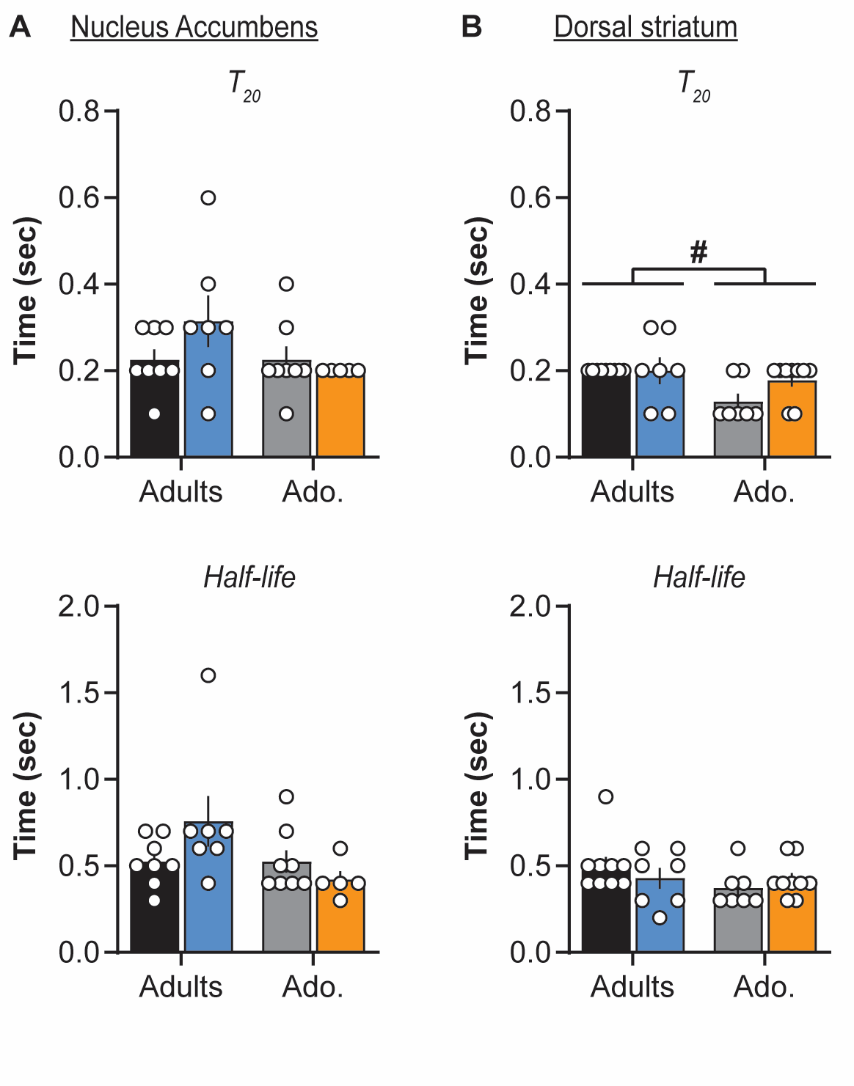
Supplementary Figure 1.** Protein restriction at adulthood or during adolescence did not change dopamine clearance parameters (T_20_: time for 20% decay from [DA]_o_ peak; Half-life: time for 50% decay from [DA]_o_ peak) after single pulse stimulation in the NAc (A; T_20_  Age F(1,24) = 2.2, *p* = 0.2; Diet F(1,24) = 0.7, *p* = 0.4; Age x Diet F(1,24) = 2.2, *p* = 0.2 / Half-life Age F(1,24) = 3.5, *p* = 0.07; Diet F(1,24) = 0.5, *p* = 0.5; Age x Diet F(1,24) = 3.5, *p* = 0.07) or in the dorsal striatum (B; T_20_  Age F(1,28) = 6.9, *p* < 0.05; Diet F(1,28) = 1.9, *p* = 0.2; Age x Diet F(1,28) = 1.9, *p* = 0.2 / Half-life Age F(1,28) = 1.9, *p* = 0.2; Diet F(1,28) = 0.04, *p* = 0.8; Age x Diet F(1,28) = 1.6, *p* = 0.2). Adults-NR (black), Adults-PR (blue), Adolescents-NR (grey) and Adolescents-PR (orange). Bars show mean ± SEM and circles show individual (*e.g.* recording site) data points. # p < 0.05 Age effect (two-way ANOVA).

**
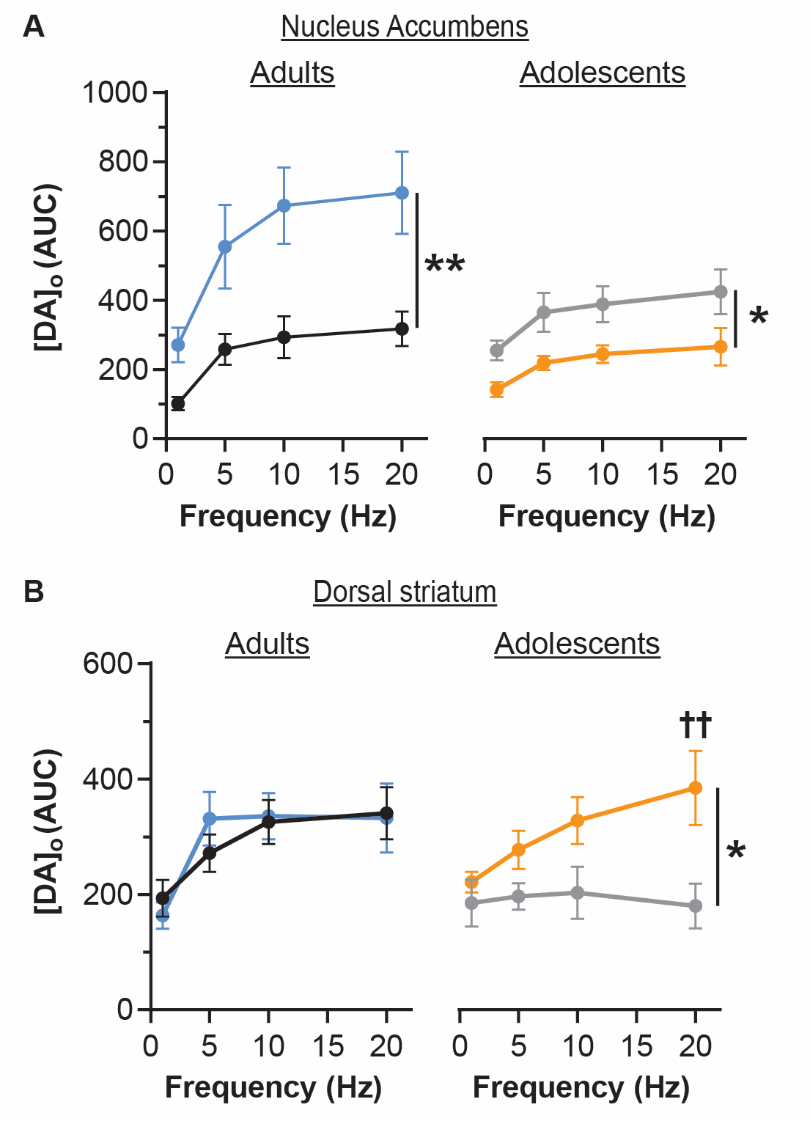
**

**Supplementary Figure 2.** Protein restriction at adulthood or during adolescence differentially altered dopamine release in the Nac and the dorsal striatum. Area under the curve (AUC) were calculated for a 5 sec window following the onset of the stimulation. (A) In the NAc, protein restriction increased frequency dependent NAc dopamine release in adult rats (*left*) but decreased it in adolescent rats (*right*). Three-way repeated measures ANOVA showed a significant effect of Frequency (F(3,72) = 39.2, *p* < 0.001), no effect of Age (F(1,24) = 3.1, *p* = 0.09) but significant interactions of Diet with other factors (Diet, F(1,24) = 1.8, *p* = 0.2; Frequency x Diet, F(3,72) = 1.6, *p* = 0.2; Age x Diet, F(1,24) = 13.4, *p* < 0.01; Frequency x Diet x Age, F(3,72) = 3.2, *p* < 0.05). Separate analyses for each Age groups confirmed the significant impact of protein restriction in both adults (two-way repeated measures ANOVA: Diet, F(1,13) = 9.3, *p* < 0.01; Frequency, F(3,39) = 43.6, *p* < 0.001; Diet x Frequency, F(3,39) = 5.3, *p* < 0.01) and adolescents (two-way repeated measures ANOVA: Diet, F(1,11) = 6.1, *p* < 0.05; Frequency, F(3,33) = 6.3, *p* < 0.01; Diet x Frequency, F(3, 33) = 0.1, *p* = 0.9). (B) In the dorsal striatum, protein restriction at adulthood did not affect dorsal striatum dopamine release in adults (*left*) but increased it in adolescent rats (*right*). Three-way repeated measures ANOVA showed a significant effect of Frequency (F(3,84) = 13.06, p < 0.001) similarly in both age groups (Age, F(1,28) = 1.5, *p* = 0.2; Frequency x Age, F(3, 84) = 2.1, *p* = 0.1), but no effect of protein restriction (Diet, F(1,28) = 3.5, *p* =0.07; Diet x Frequency, F(3,84) = 1.8, *p* = 0.1; Diet x Age, F(1,28) = 2.6, *p* = 0.1; Diet x Frequency x Age, F(3,84) = 1.9, *p* = 0.1). Separate analyses for each age group confirmed no effect of protein restriction in adults (two-way repeated measures ANOVA: Diet, F(1,14) = 0.02, *p* = 0.9; Frequency, F(3,42) = 26.6, *p* < 0.001; Diet x Frequency, F(3, 42) = 1.8, *p* = 0.2), but demonstrated a significant increase of evoked dopamine release after protein restriction during adolescence (Diet, F(1,14) = 8.7, *p* < 0.05; Frequency, F(3,42) = 1.8, *p* = 0.2; Diet x Frequency, F(3, 42) = 1.9, *p* = 0.1). Values are represented as mean ± SEM. * *p* < 0.05, ** *p* < 0.01 Diet effect (two-way ANOVA), †† *p* < 0.01 Frequency effect (two-way ANOVA followed by Dunnett’s *post hoc* tests versus 1 Hz).
